# Phosphorylation of extracellular signal-regulated kinase as a biomarker for cannabinoid receptor 2 activation

**DOI:** 10.1016/j.heliyon.2018.e00909

**Published:** 2018-11-07

**Authors:** Jingru Wang, Juehua Xu, Yanyan Peng, Yue Xiao, Huang Zhu, Zhi-Ming Ding, Haiqing Hua

**Affiliations:** Lilly China Research and Development Center (LCRDC), Eli Lilly & Company, Shanghai, 201203, China

**Keywords:** Cell biology, Immunology

## Abstract

Cannabinoid receptor 2 (CB2R) is a therapeutic target in inflammatory diseases; its activation by agonists provides important clinical information, but there are currently no methods to quantify CB2R activation in humans. Chinese hamster ovary (CHO)-K1 cells and mouse and human whole blood cells were used for experiments. CB2R was activated in cells by treatment with the agonist CP55,940. Cells were also pretreated with proprietary Compound A and B (experimental agonists). We developed our method based on the finding that CB2R ligand binding and activation stimulates acute-phase extracellular signal-regulated kinase (ERK) phosphorylation in human and rodent immune cells, after which CB2R becomes unresponsive to stimulation by a second CB2R agonist CP55940 for a certain time period. We detected ERK phosphorylation as a measure of target engagement in mouse and human whole blood cells by flow cytometry. In cells overexpressing human or mouse CB2R, pretreatment with Compound A dose-dependently inhibited ERK phosphorylation for 2 h, prolonging the time window for measuring ERK phosphorylation. Our method enables measurement of CB2R activation by its agonists in human blood cells based on detection of ERK phosphorylation, which is useful for therapeutic drug monitoring and other clinical applications.

## 1 Introduction

The endocannabinoid system comprises natural ligands (endogenous endocannabinoids such as 2-arachidonoylglycerol [2-AG]), enzymes for endocannabinoid biosynthesis and degradation, and type 1 and 2 cannabinoid receptors (CB1R and CB2R, respectively). CB1R is widely expressed in the nervous system and regulates a broad range of biological functions such as energy homeostasis by suppressing appetite and nutrient transport and increasing energy expenditure [1]. The endocannabinoid system mediates both immunosuppressive and anti-inflammatory. Although both CB1 and CB2 receptors have been detected in leukocyte mediators of inflammation, CB2 is believed to play a more important role and appears to be the key mediator of cannabinoid regulation of inflammation and immune functions. CB2R is primarily expressed in peripheral organs and especially in immune cells [2, 3]: compared to CB1R, it shows 10- to 100-fold higher expression in cells of the immune system such as B and T lymphocytes, monocytes, and macrophages [4]. CB2R plays an important role in regulating inflammatory responses under both physiological and pathological conditions, and small-molecule modulators of CB2R including agonists have been developed for the treatment of various inflammatory diseases [5, 6]. Blockade of the CB2 receptor with CB2 antagonist SR145528 inhibits splenocyte proliferation and induces apoptosis in vitro [7]. In macrophages, CB2 stimulation suppresses proliferation and the release of cytokines, such as IL-2, IL-12 and TNF-α [8]. Activation of CB2 induces natural killer cell migration and suppresses neutrophil migration and differentiation [9]. Therefore, selective CB2 receptor activation has the potential to provide the anti-inflammatory effects of cannabinoids without the psychoactive effects, caused by CB1 activation.

When testing a receptor agonist in clinical trials, it is useful to have a biomarker that serves as an indicator of receptor activation level for hypothesis validation [10]. However, for a certain class of therapeutic targets such as G protein-couple receptors (GPCRs), it is a challenge to detect receptor activation in human subjects, partly due the complexity of the downstream signaling [11]. Upon activation, CB2 triggers both ERK and AKT signaling. The cAMP level is also reduced by CB2. CB2 recruits β-arrestins upon activation by agonists. β-Arrestin recruitment is induced by phosphorylation of their C-terminal tails, and is associated with the termination of GPCR signaling. Despite the understanding of CB2 mediated signaling, to date, there have been no methods developed for clinical testing of CB2R activation. To this end, in this study we evaluated all the downstream signaling of CB2 and developed a method for quantifying CB2R activation in primary mouse and human blood cells based on the findings that (1) CB2R activation results in phosphorylation of extracellular signal-regulated kinase (ERK) in blood cells; and (2) CB2R agonist (e.g. Compound A) causes cells to be less responsive to stimulation by a second agonist (e.g. CP55,940). We used this method to detect the of CB2R in response to an agonist in vitro and in vivo, and demonstrate that it can potentially be applied to human subjects in clinical studies.

## 2 Materials and methods

### 2.1 Cell culture

Chinese hamster ovary (CHO)-K1 cells were obtained from the American Type Culture Collection (Manassas, VA, USA) and used to transiently express human and mouse CB2R. The cells were maintained in Ham's medium containing 10% fetal calf serum, 100 U/ml penicillin/streptomycin, and 2 mM l-glutamine at 37 °C in 5% CO_2_/95% air.

### 2.2 Human whole blood cell culture

Human whole blood cells were purchased from commercial sources (Allcells, Alameda, CA, USA or Zen-Bio, Research Triangle Park, NC) and were cultured in Roswell Park Memorial Institute (RPMI)-1640 medium containing 2 mM l-glutamine, 10% fetal bovine serum, and 100 U/ml penicillin/streptomycin at 37 °C in 5% CO_2_/95% air.

### 2.3 Cell-based quantitative detection of AKT and ERK phosphorylation

CHO-K1 cells transiently expressing human or mouse CB2 receptor were seeded in 96-well plates and cultured overnight. The next day, the cells were starved for 2 h in Dulbecco's Modified Eagle's Medium or RPMI-1640 medium containing 0.1% bovine serum albumin. Compounds at various concentrations were then added and the cells were incubated for indicated time periods at 37 °C. Phosphorylated AKT levels were measured using the cyclic (c)AMP Cellular Assay and Phospho-AKT (Ser473) Cellular Assay kits (both from Cisbio, Bedford, MA, USA), and phosphorylated (p-)ERK levels were measured using the Phospho-ERK (Thr202/Tyr204) Cellular Assay kit (Cisbio) according to the manufacturer's protocol (time resolved fluorescence technology). The experiments were performed in triplicates and the data were analyzed using Prism Graphpad (four parameter curve fitting and one-way ANOVA).

### 2.4 Western blots

CHO cells were stimulated with CB2 agonist CP55,940 and incubated at 37 °C for indicated time and then the cells were pelleted and lysed. With the lysis in RIPA with the RIPA lysis buffer and centrifugation for 20 min, proteins were extracted from cells. Bradford method was used to detect the quality of the proteins. SDS-PAGE was used to separate the proteins with equal amount, the proteins were then transferred onto PVDF membrane, with the primary antibodies, all purchased from Cell Signaling, including Phospho-p44/42 MAPK (Erk1/2) Thr202/Tyr204 antibody (Cat. No. 4370), Phospho-AKT (Ser473) antibody (Cat. No. 4060), Phospho-SAPK/JNK (Thr183/Tyr185) antibody (Cat. No. 9255) and Phospho-p38 MAPK (Thr180/Tyr182) (28B10) antibody (Cat. No. 9216) at 4 °C for 24 h. β-actin acted as internal control. Then the membrane was washed and incubated with the secondary antibodies at room temperature for another 1 hour. The image of the membrane was acquired using iBind Flex system (ThermoFisher Scientific).

### 2.5 Fluorescence-activated flow cytometry analysis of mouse and human whole blood

C57BL/6J mice were purchased from LinChang Shanghai (Shanghai, China) and experiments were carried out in the ChemPartner animal facility (Shanghai, China). All procedures involving animals were conducted humanely by or under the direction of highly trained and experienced personnel. The protocol was reviewed and approved by the Institutional Animal Care and Use Committee of ChemPartner and the review team of Eli Lilly & Company. Fluorescein isothiocyanate-conjugated mouse anti-cluster of differentiation (CD)3 antibody and allophycocyanin-conjugated mouse anti-CD19 antibody were diluted 1:10 in mouse or human whole blood. Blood (180 μl/well) was added to 96-well plates and incubated at 37 °C for 60 min. A 20-μl volume of 10× compound stock solution was added to the blood in each well, followed by incubation at 37 °C for 5 min. The mixture was then transferred to 96-well deep-volume plates and Lyse/Fix buffer was added to each tube. After incubating at 37 °C for 15 min, the cells were centrifuged at 600 × *g* for 5 min at room temperature and washed once with 1.5 ml phosphate-buffered saline (PBS) before cold BD Phosflow Perm Buffer was added (BD Biosciences, Franklin Lakes, NJ, USA). After incubation on ice for 30 min, the cells were washed twice with 1 ml stain/wash buffer. Anti-phospho-p44/42 mitogen-activated protein kinase (ERK1/2) (Cell Signaling, Cat. No. 4370), antibody was diluted in the stain/wash buffer (400×) and the cells were incubated in this solution overnight at 4 °C. The following day, the cells were washed once with 1 ml stain/wash buffer. Phycoerythrin-conjugated anti-rabbit IgG was diluted in stain/wash buffer (400×) and the cells were incubated in this solution for 60 min at room temperature. After washing once with 1 ml stain/wash buffer, the cells were resuspended in 400 μl PBS buffer and analyzed by flow cytometry. Fluorescence-activated cell sorting data were analyzed with FlowJo v.10 software (Tree Star, Ashland, OR, USA). For data analysis, CD3^+^ and CD19^+^ cells were gated as T and B cells, respectively. Results are shown as % pERK-positive cells of all gated cells. Mean differences were evaluated by analysis of variance. The experiments were performed in triplicates and the data were analyzed using Prism Graphpad (four parameter curve fitting and one-way ANOVA).

### 2.6 Ethical approval

All procedures performed in studies involving animals were in accordance with the ethical standards of the institution or practice at which the studies were conducted. The protocol was reviewed and approved by the Institutional Animal Care and Use Committee of ChemPartner and the review team of Eli Lilly & Company.

## 3 Results

### 3.1 2-AG activates multiple signaling pathways downstream of CB2R including AKT and ERK

**Fig. 1:**
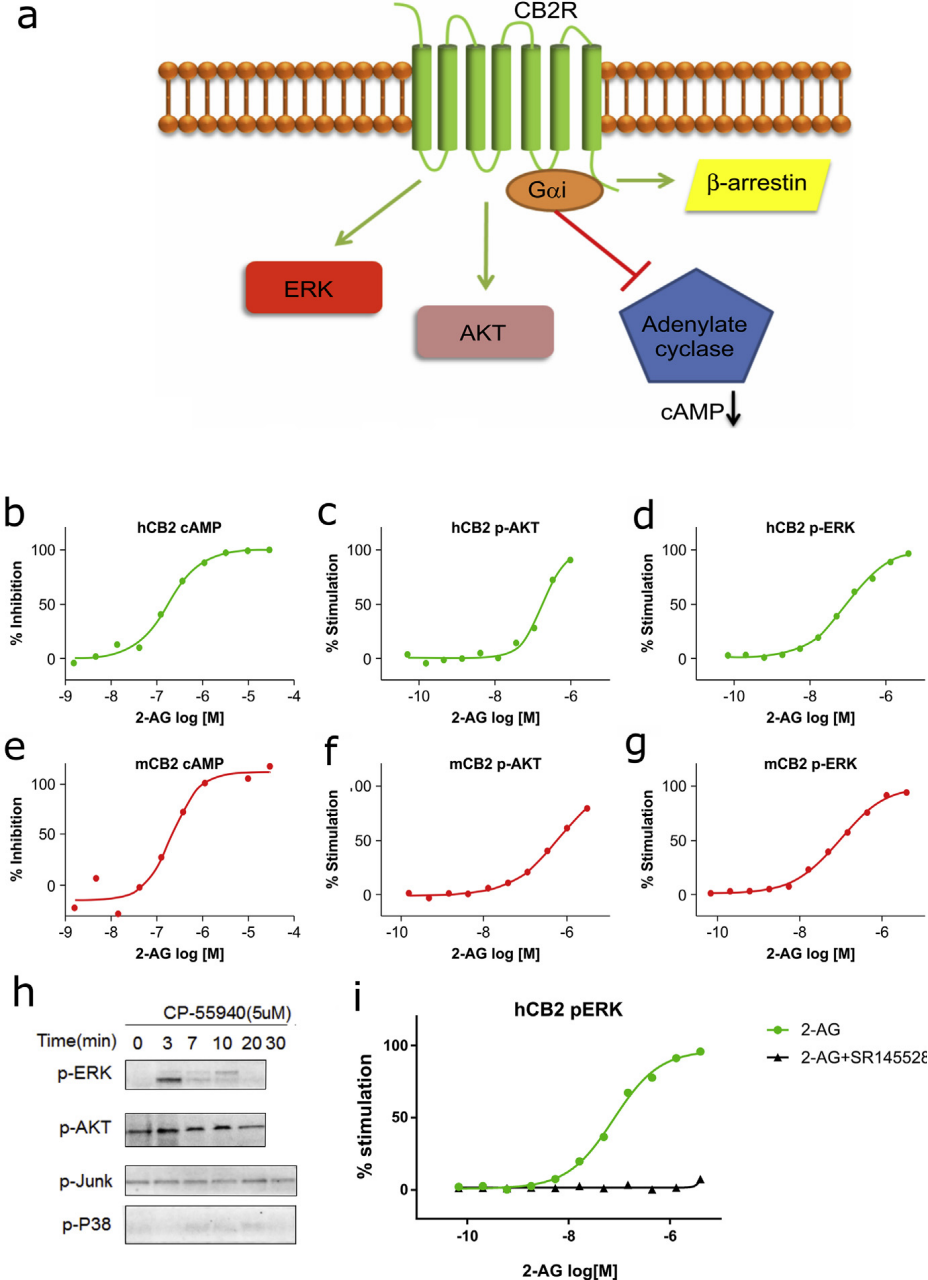
(a) CB2R activation activates multiple downstream signaling pathways. Signaling events induced by CB2R activation include changes in β-arrestin, cAMP, ERK, and AKT. 2-AG dose-dependently activated Gαi signaling (inhibition of cAMP) and induced AKT and ERK phosphorylation in cells overexpressing human CB2R (b–d) and mouse CB2R (e–g). Experiments were performed three times and representative plots are shown. Phosphorylation of ERK, AKT, JNK and P38 in CHO cells after CP55,940 treatment determined by western blot (h). ERK phosphorylation upon CB2 activation with and without 1 uM SR145528 (i).

Biomarkers of receptor activation are used in clinical investigations to monitor the pharmacodynamic effects of receptor modulators. Along with downstream effectors, multiple signaling pathways can be activated by a surface receptor. In order to develop a method that can be used to monitor the effects of CB2R agonists, we detected the expression of proteins downstream of CB2R including β-arrestin, and Gαi as well as AKT and ERK phosphorylation (Fig. 1a). For a biomarker of receptor activation to be useful, its signal intensity must correlate with the dose (concentration) of the receptor agonist. In CHO cells overexpressing either human or mouse CB2R, we were able to detect levels of cAMP (Fig. 1b, e) along with phosphorylation of AKT (Fig. 1c, f) and ERK (Fig. 1d, g, h; Fig. S1). The ERK phosphorylation signal was blocked by adding a CB2R antagonist SR145528 (Fig. 1i).

### 3.2 CB2R activation triggers ERK phosphorylation in primary B cells

**Fig. 2:**
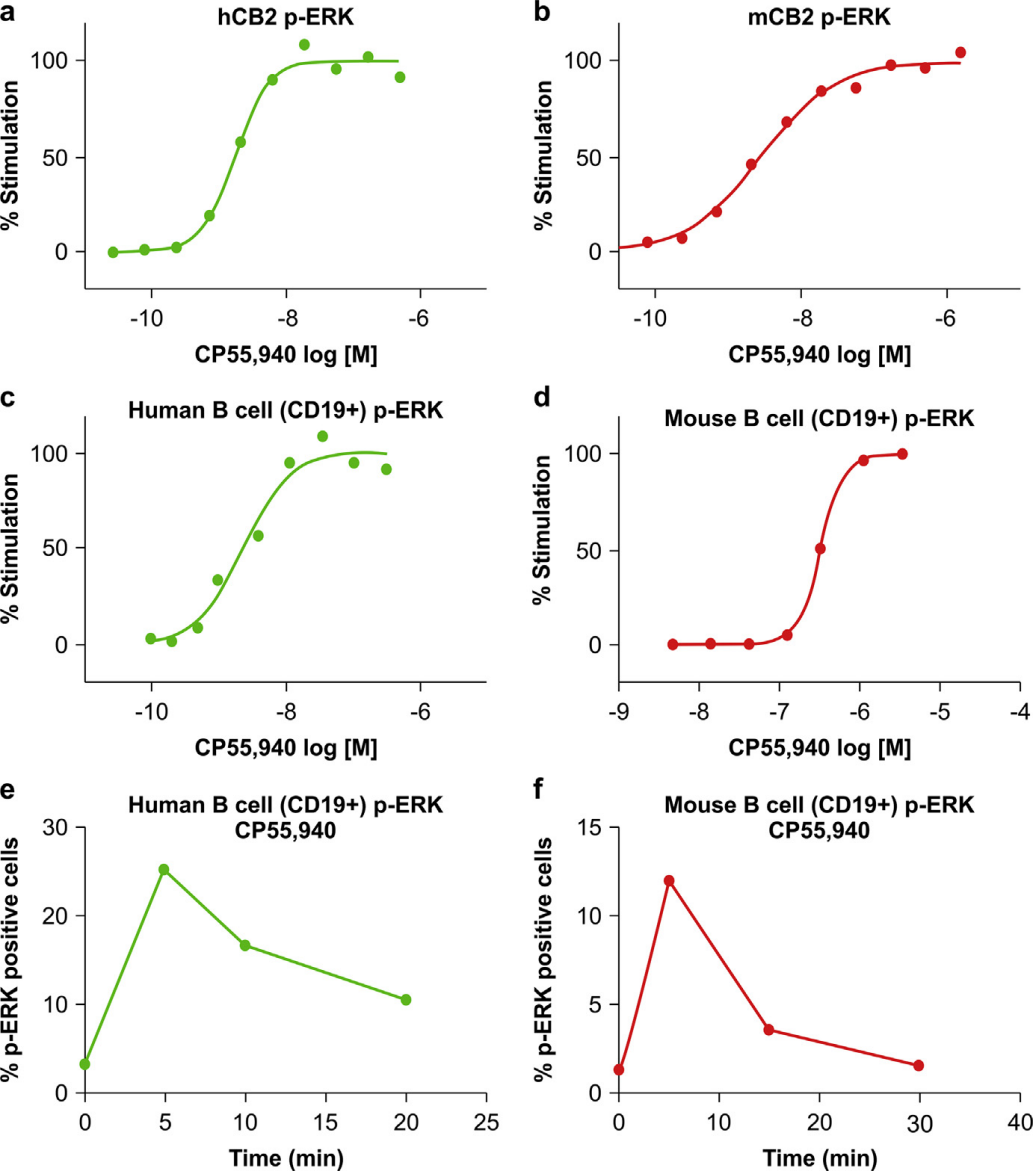
ERK phosphorylation induced by CB2R agonist in mouse whole blood. (a,b) ERK phosphorylation following CP55,940 treatment in CHO cells overexpressing CB2R. (c–f) Dose-dependent (c,d) and time-dependent (e,f) ERK phosphorylation following CP55,940 treatment in B cells from peripheral blood. Experiments were performed three times and representative plots are shown.

For clinical application, the biomarker signal must be robust and stable in primary cells. After evaluating the signaling molecules downstream of CB2R, we found that ERK phosphorylation was the most suitable biomarker in primary cells. We therefore evaluated ERK phosphorylation resulting from CB2R activation induced by the synthetic agonist CP55,940 [9]. In CHO cells overexpressing human or mouse CB2R, CP55,940 dose-dependently induced ERK phosphorylation (Fig. 2a, b). Since CB2R is highly expressed in immune cells, we evaluated ERK phosphorylation in human or mouse B cells (CD19^+^) from peripheral blood treated with CP55,940 and obtained the same result as in CHO cells (Fig. 2c, d). We also found that ERK phosphorylation is a time-dependent event, with the signal peaking within 5–10 min of CP55,940 treatment (Fig. 2e, f).

### 3.3 Indirect measurement of CB2R activation based on ERK phosphorylation

**Fig. 3:**
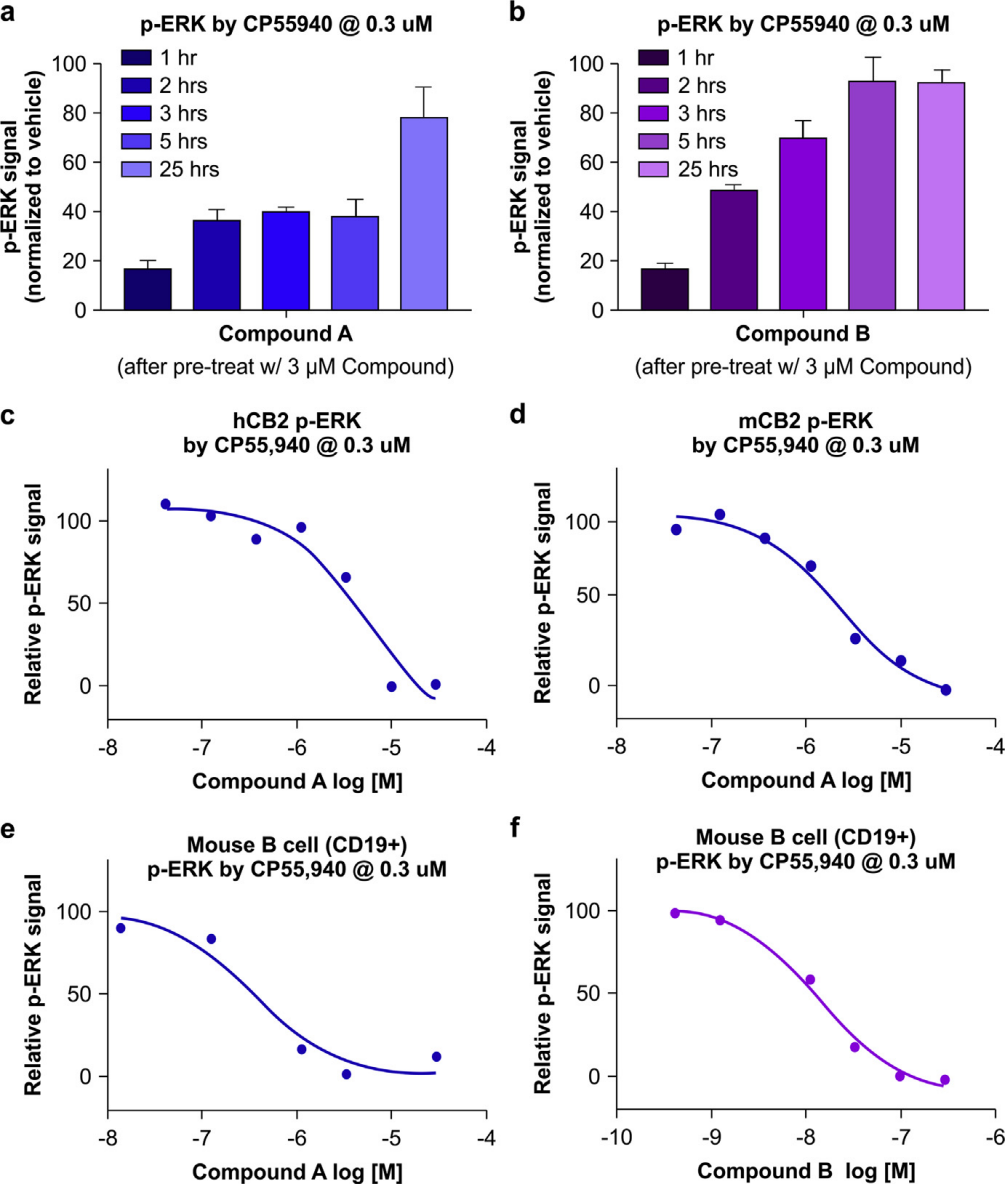
ERK phosphorylation indirectly measures CB2R activation by agonists. (a,b) Time-dependent resensitization of CB2R measured by ERK phosphorylation after treatment with 0.3 μM CP55,940 in CHO cells overexpressing CB2R. Cells were pretreated with 3 μM Compound A or B. (c–f) Pretreatment with Compound A dose-dependently suppresses the pERK signal induced by CP55,940 in CHO cells overexpressing human or mouse CB2R (c,d) and in mouse B cells (e,f). Experiments were performed three times. Standard error of the means and representative plots are shown.

Although we observed that CB2R activation (by CP55,940) caused ERK phosphorylation in primary B cells, this effect was transient and the signal declined after about 30 min. This poses a major challenge for in vivo application in animals or humans administered an agonist, since drawing blood 5–10 min after treatment for immediate measurement of ERK phosphorylation is not feasible in a clinical setting. We therefore monitored CB2R activation in response to two different experimental agonists (hereafter referred to as Compounds A and B) over a longer time frame than 30 min. The pharmacological profiles of the two agonists (Compound A and B) are shown in Table 1. Both compounds induced β-arrestin recruitment, which is known to regulate internalization and resensitization of GPCR [13]. We reasoned that pretreatment with Compound A or B to mimic in vivo dosing with the compounds would result in reduced ERK phosphorylation in CB2R-expressing cells during the receptor resensitization period. We tested this hypothesis in CHO cells and confirmed that pretreatment with 3 μM Compound A or B caused the cells to become unresponsive to CP55,940 (0.3 μM) at the 1-h time point (Fig. 3a, b). The responsiveness to CP55,940, as measured by ERK phosphorylation, gradually recovered within 25 h. This effect was correlated with the dose of CB2R agonist (Compound A). In cells overexpressing human or mouse CB2R, pretreatment with Compound A dose-dependently inhibited ERK phosphorylation 2 h later (Fig. 3c, d). Thus, this method increases the time window for measuring ERK phosphorylation. We tested this method in mouse primary B cells and found that consistent with the above findings, both Compounds A and B dose-dependently inhibited CP55,940-induced ERK phosphorylation at 2 h.

**Table 1:** CB2 agonists used in this study.

Agonist	Binding K_i_ (nM)	β-Arrestin EC50 (nM)
CP55,940	1^b^	4.9
Compound A^a^	9	5.5
Compound B^a^	N/A	6.2

a Compound A and B are proprietary CB2 agonists.

b Public information [12].

### 3.4 Detection of CB2R activation in vivo

**Fig. 4:**
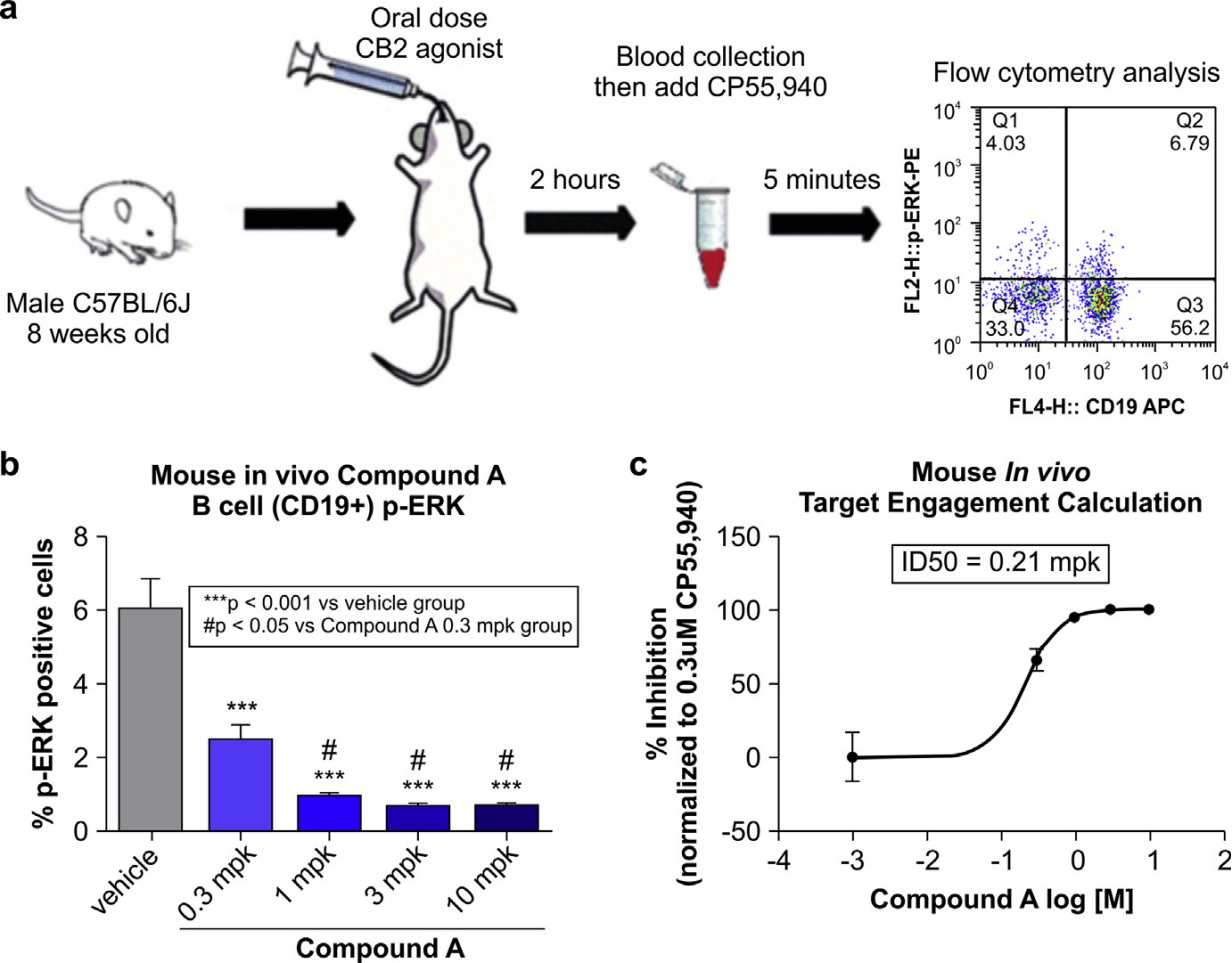
Detection of CB2R activation in mice by indirect measurement of ERK phosphorylation. (a) Schematic illustration of the procedure for measuring CB2R activation in mouse. (b) Concentration-dependent inhibition of CP55,940-induced ERK phosphorylation in B cells ex vivo by in vivo dosing with Compound A (mpk: mg compound per kg body weight). (c) Quantification of Compound A target engagement with CB2R, as indicated by percent inhibition of ERK phosphorylation. “Target engagement” refers to the extent of CB2R activation induced by Compound A relative to its full activation status, which is indicated by percent inhibition of ERK phosphorylation. Error bars represent standard deviation. ***P < 0.001 vs. vehicle group; ^#^P < 0.05 vs. Compound A (one-way analysis of variance).

To determine whether our method is applicable to clinical settings, we developed a procedure to measure CB2R activation in vivo. Mice were orally dosed with CB2R agonist, and 2 h later whole blood was drawn and treated with CP55,940 before ERK phosphorylation was detected by flow cytometry (Fig. 4a). We found that administration of Compound A dose-dependently inhibited ERK phosphorylation induced by CP55,940 ex vivo (Fig. 4b). Based on these results, we calculated the target engagement of Compound A and determined that at 0.21 mpk (mg compound per kg body weight) the efficacy was 50% whereas above 3 mpk, Compound A fully engaged the target CB2R (Fig. 4c).

### 3.5 Clinical application of ERK phosphorylation as a biomarker for CB2R activation

**Fig. 5:**
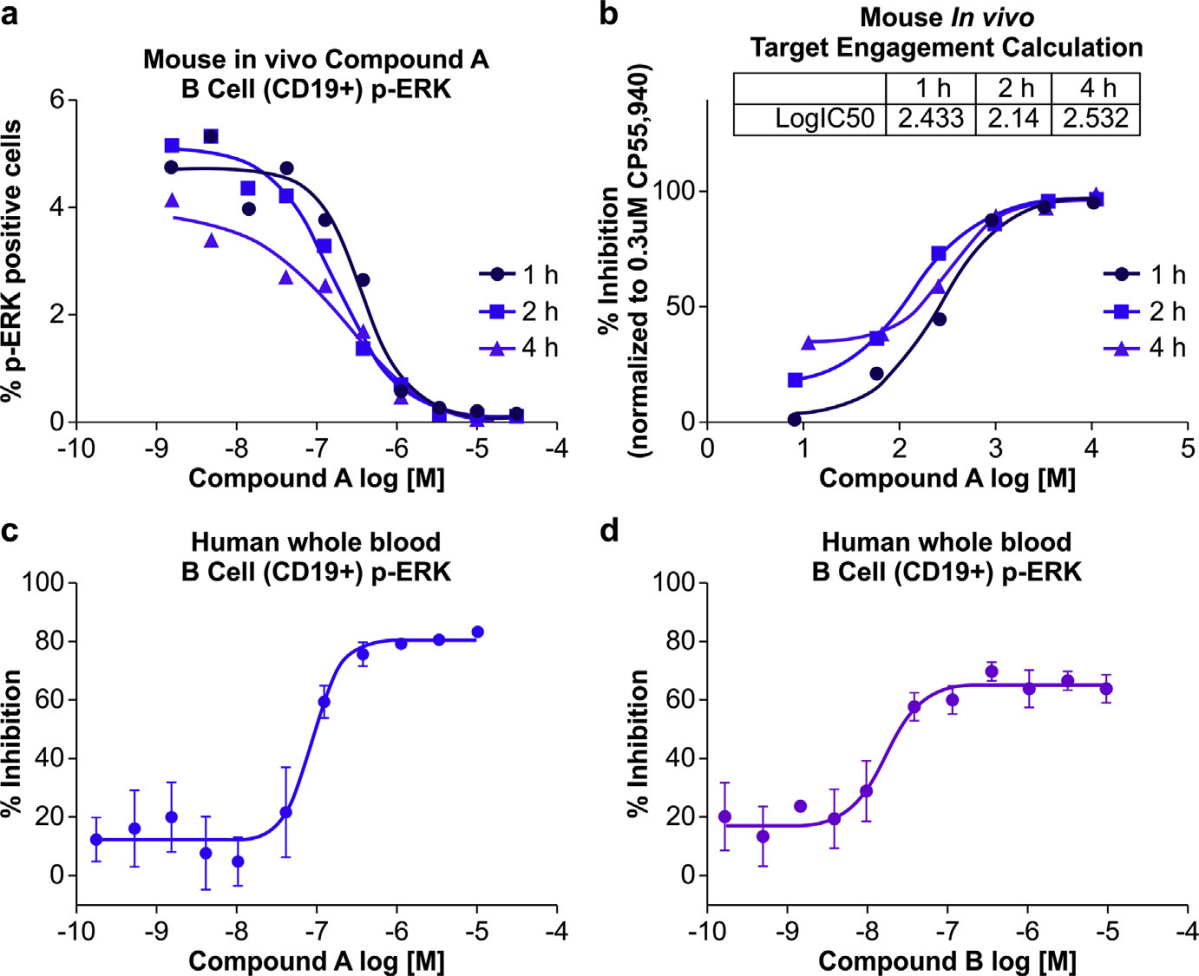
Detection of CB2R activation in human whole blood by indirect measurement of ERK phosphorylation. (a) Concentration-dependent inhibition of CP55,940-induced ERK phosphorylation in mouse B cells treated with Compound A for indicated times (1, 2, and 4 h). (b) Quantification of Compound A target engagement with CB2R, as indicated by percent inhibition of ERK phosphorylation. (c,d) Pretreatment with Compound A or Compound B dose-dependently suppressed the pERK signal induced by CP55,940 in B cells from human whole blood. Error bars represent standard deviation.

It is useful if in laboratories blood samples can be stored at room temperature for a short period of time if they cannot be immediately processed. We therefore modified the procedure such that we allowed the whole blood samples to stand at room temperature for 1, 2, and 4 h after they were drawn before adding CP55,940. The CP55,940 was added to whole blood and 5 min later, the blood cells were used for ERK phosphorylation analysis. We found that the ERK phosphorylation signal was inhibited to the same extent at the different time points (Fig. 5a) and that the calculated CB2R activation levels were also comparable (Fig. 5b). This indicated that stable measurements can be obtained by our method even when blood samples are not freshly drawn, which is an advantage in clinical settings. We also tested our method with human whole blood pretreated with Compounds A and B to mimic dosing of human subjects. After 2 h, CP55,940 was added and ERK phosphorylation was measured. Similar to the results obtained with mouse blood, we found that Compound A and B dose-dependently inhibited ERK phosphorylation induced by CP55,940 (Fig. 5c, d).

## 4 Discussion

CB2R is a G protein-coupled receptor that activates multiple downstream signaling pathways; measurement of these signaling events can be used to quantify CB2R activation. β-Arrestin is recruited to activated CB2R [14]. In primary cells, it is technically challenging to measure the physical interaction between β-arrestin and CB2R. Gαi protein is coupled to CB2R and upon activation, inhibits adenylyl cyclase and subsequently reduces cAMP level [15]. In order to detect this decrease, cells are typically treated with forskolin to increase the baseline cAMP level. However, forskolin has variable effects in primary cells; moreover, there is a limited time window for detecting the decline in the cAMP signal following CB2 activation in human primary cells. In the present study, we detected CB2R activation in human primary cells by evaluating AKT and ERK phosphorylation. The phosphorylation of ERK resulting from CB2R activation was more robust and stable than that of AKT, and was therefore used to monitor CB2 activation. Using either homogenous time-resolved fluorescence- or flow cytometry-based technology, we were able to detect ERK phosphorylation in mouse and human CD19^+^ B cells. However, since the pERK signal declines so rapidly following receptor activation, using ERK phosphorylation as a measure of CB2R activation is impractical in vivo.

We circumvented this problem by instead indirectly measuring pERK level. At 2 h after oral administration of an experimental agonist (e.g., Compound A), primary B cells in the blood became less sensitive to stimulation by a second CB2R agonist (e.g., CP55,940); this resulted in a weaker pERK signal that was then converted into a measure of CB2R activation, which is proportional to engagement of the agonist. Since ERK phosphorylation is correlated with CB2R activation in terms of both intensity and time, we believe that ERK phosphorylation is closely associated with CB2R activation and that our method faithfully recapitulates the activity of the CB2R agonist. The ability to determine the relative activity of agonists is clinically valuable for dosage adjustment in therapeutic regimens for the treatment of inflammatory diseases.

In conclusion, we developed a method that enables measurement of CB2R activation by its agonists in human blood cells. This method facilitates the study of CB2R agonists and their mechanisms of action, although it may require refinement before it can be adapted to specific clinical applications.

## Declarations

### Author contribution statement

Jingru Wang: Conceived and designed the experiments; Performed the experiments; Analyzed and interpreted the data; Contributed reagents, materials, analysis tools or data; Wrote the paper.

Juehua Xu, Yanyan Peng, Yue Xiao, Huang Zhu: Performed the experiments.

Zhi-Ming Ding: Analyzed and interpreted the data.

Haiqing Hua: Conceived and designed the experiments; Analyzed and interpreted the data; Wrote the paper.

### Funding statement

This work was supported by Eli Lilly & Co.

### Competing interest statement

The authors declare the following conflicts of interest: All authors are employees of and may hold stocks for Eli Lilly & Co., which provided support in the form of salaries for authors and funding for research materials, but did not have any additional role in study design, data collection and analysis, decision to publish, or preparation of the manuscript.

### Additional information

No additional information is available for this paper.

## Appendix A Supplementary data

Supplementary Gel Images **Fig. S1:** Phosphorylation of ERK, AKT, JNK and P38 in CHO cells after CP55,940 treatment determined by western blot. Supplementary Gel Images
