# Supplementary material for: Phosphorylation of extracellular signal-regulated kinase as a biomarker for cannabinoid receptor 2 activation
Source: Heliyon. 2018 Nov 7;4(11):e00909. doi: 10.1016/j.heliyon.2018.e00909 (PMC6226583; doi:10.1016/j.heliyon.2018.e00909)
Supplement: Supplementary Gel Images — Fig. S1: Phosphorylation of ERK, AKT, JNK and P38 in CHO cells after CP55,940 treatment determined by western blot. [file mmc1.pptx]

## Slide 1
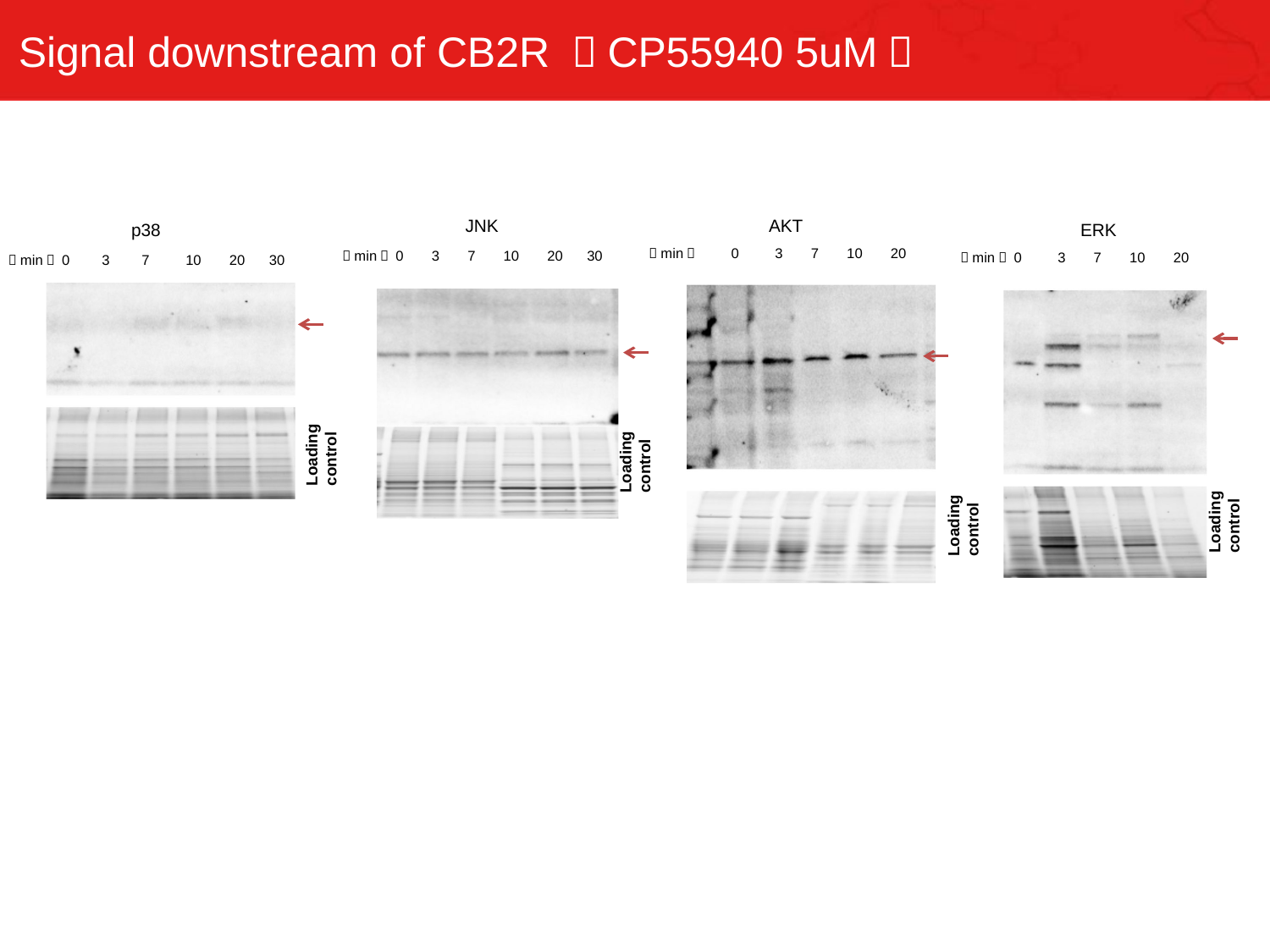

# Signal downstream of CB2R （CP55940 5uM）
JNK
（min） 0 3 7 10 20 30
AKT
（min） 0 3 7 10 20
p38
（min） 0 3 7 10 20 30
ERK
（min） 0 3 7 10 20
Loading
control
Loading
control
Loading
control
Loading
control
